# SOCIAL NETWORK SIZE, SOCIAL FULFILLMENT, AND FINANCIAL EXPLOITATION VULNERABILITY IN OLDER ADULTS

**DOI:** 10.1093/geroni/igae098.3145

**Published:** 2024-12-31

**Authors:** Daisy Noriega-Makarskyy, Shaneen Upal, Elizabeth Erdman, Laura Fenton, Aaron Lim, Gali Weissberger, Laura Mosqueda, Duke Han

**Affiliations:** University of Southern California, Los Angeles, California, United States; University of Southern California, Los Angeles, California, United States; University of Southern California, Los Angeles, California, United States; University of Southern California, California, United States; University of Southern California, Los Angeles, California, United States; Bar-Ilan University, Ramat Gan, HaMerkaz, Israel; University of Southern California Keck School of Medicine, Pasadena, California, United States; University of Southern California, Los Angeles, California, United States

## Abstract

This study tested whether aspects of social functioning, including breadth of current social roles (e.g., being a family member, co-worker, neighbor, etc.), and fulfillment in various social supports (i.e., relationship depth), are associated with perceived financial exploitation vulnerability (FEV) among older adults. Adults aged 50 or older (N=115; 69% of sample >65 years) completed a laboratory visit consisting of questionnaires assessing relationship breadth, depth, and perceived FEV. The relationship depth measure included a total score as well as subscales for: 1) Appraisal, or availability of interpersonal advice or guidance; 2) Belonging, or availability of interpersonal empathy and acceptance; and 3) Tangible, or material/financial support resources. Hierarchical linear regressions separately tested associations between relationship depth or breadth and FEV. After covarying for demographics, depression/anxiety symptoms, and general cognition, FEV was not significantly associated with relationship breadth, or the Appraisal and Tangible subscales (p’s=.13-.93). FEV was negatively associated with Relationship Depth Total Score (B(SE) = -.08(.03), p<.01) and Belonging subscale (B(SE) = -.28(.06), p<.001). In summary, among a sample of older adults, perceived financial exploitation vulnerability was not associated with the number of different regular social roles a person had, or the availability of social resources for material/financial assistance, or interpersonal advice/guidance. Rather, financial exploitation vulnerability was greatest among individuals with a weaker sense of social belonging, marked by instances such as not having someone to go with on a spontaneous day trip. Such results corroborate the benefits of close interpersonal relationships that may reduce vulnerability to financial exploitation in older age.

